# Unbiased combination screening on repurposed drugs reveals synergistic potential of copanlisib and cerivastatin against chemoresistant high-grade serous ovarian cancer

**DOI:** 10.1186/s13048-025-01828-7

**Published:** 2025-11-06

**Authors:** Yu Sun, Yangyang Wang, Syed Umbreen, Benjamin Pepperrell, Niamh Buckley, Paul Mullan, Ahlam Ali, Fiona Furlong

**Affiliations:** 1 School of Pharmacy, Queen’s University Belfast, Belfast, Northern Ireland; 2 Anhui Institute of Medicine, Hefei, China; 3 North-South Research Program, The All-Ireland Cancer Liquid Biopsies Consortium (CLuB), Dublin, Ireland; 4 Patrick G. Johnson Centre for Cancer Research, Queen’s University Belfast, Belfast, Northern Ireland; 5 School of Life Sciences, Faculty of Science and Engineering, Department of Biomedical Science and Physiology, University of Wolverhampton, Wolverhampton, UK

**Keywords:** Drug screening, Drug combinations, Chemoresistant high-grade serous ovarian cancer

## Abstract

**Introduction:**

High-grade serous ovarian cancers (HGSOCs) are challenging to treat and often resistant to therapy. Despite ongoing therapeutic progress, relapse and poor outcomes remain common among both newly diagnosed and recurrent cases. Systematic high-throughput screening of clinically approved compounds holds significant promise for uncovering novel therapeutic responses and developing new treatment strategies for this disease.

**Methods:**

A panel of drugs were screened for cytotoxicity in five HGSOC cell lines, with drug efficacy quantified using the drug sensitivity score (DSS). All pairwise combinations of 384 low-cytotoxic drugs were screened by grouping 10 compounds in each well. The potent 10-compound combinations were deconvoluted into 2-drug pairings for secondary screening and ranked by the Bliss independent model and the Loewe additive model. Promising drug responses were further characterised in 3D spheroid cultures and patient ascites-derived cells (PADCs). The mechanism of action of the drugs was investigated by Western blot analysis.

**Results:**

The DSS profile of drug responses provided a more robust clustering of 5 HGSOC cell lines according to their chemosensitivity responses compared to gene expression analysis of chemoresistance markers. Furthermore, chemoresistant HGSOC cell lines exhibited limited efficacy to single-agent treatments and generally demonstrated resistance to most anti-cancer agents. However, combination screens identified several novel drug pairings that were cytotoxic to chemoresistant HGSOC cells. Drug combinations involving traditional anticancer agents showed superior synergy and toxicity in chemosensitive cell lines, while all cell lines demonstrated good sensitivity to PI3K and HMG-CoA reductase inhibitors at sub-maximal clinically relevant concentrations, with the greatest sensitivity observed in chemoresistant cells. The combination of PI3K and HMG-CoA reductase inhibition significantly reduced the viability and growth of HGSOC spheroids. PADCs exhibited intrinsic sensitivity to HMG-CoA inhibition, while the combination with PI3K inhibition facilitated further dose reductions. Mechanistic studies revealed that the HMG-CoA inhibitor increased phospho-Akt levels in chemoresistant cell lines, sensitising them to PI3K inhibition.

**Conclusion:**

This study demonstrates the application of multiplex drug combination screening to identify effective synergistic therapies. Co-targeting PI3-kinase and HMG-CoA reductase could be repurposed as a potent combination to treat chemoresistant HGSOC.

**Supplementary Information:**

The online version contains supplementary material available at 10.1186/s13048-025-01828-7.

## Introduction

Ovarian cancer (OC) is the most lethal gynaecologic malignancy worldwide, with over 60% of patients relapsing within five years of initial chemotherapy [1]. High-grade serous ovarian cancer (HGSOC), accounting for 70–80% of cases, is particularly aggressive due to frequent homologous recombination deficiencies (HRD) and rapid development of chemoresistance [2, 3]. Although most HGSOC patients respond well to platinum-based chemotherapy with clinical remission, over 80% will relapse with chemoresistance and ultimately die from their disease [4]. Recent advances in targeted therapies, including PARP inhibitors (e.g., olaparib, niraparib) and antiangiogenics (e.g., bevacizumab), have improved progression-free survival in select subgroups (mostly patients with positive HRD), yet durable responses remain elusive for most patients.

Given the limited efficacy of monotherapy and its potential to induce resistance, combination therapies remain a cornerstone strategy to offer diverse treatment options and to improve overall outcomes. Several clinical trials combined DNA damage checkpoint inhibitors or antiangiogenics with standard chemotherapy in platinum-resistant HGSOC. Combinations of gemcitabine with adavosertib [5], berzosertib [6], or bevacizumab [7] produced modest benefits in progression-free survival (PFS) with reported improvements ranging from approximately 1 to 3 months, however, improvements in overall survival (OS) were not generally achieved. Regarding acquired resistance to PARP inhibitors, common due to their widespread use in maintenance therapy, only one combination, prexasertib plus olaparib, was evaluated in a clinical trial. Although it yielded a partial response rate of 30.6%, the regimen was associated with significant toxicity (≥ 10% adverse events) [8]. Therefore, more effective treatment for ovarian cancer patients, especially patients who develop chemoresistance and drug-refractory disease, is increasingly critical, highlighting the need for more personalised therapy options to enhance the quality of life for patients living with OC.

Better drugs to treat OC may already exist. Drug repurposing, the process of identifying new indications for existing drugs, offers a cost-effective, time-efficient, and lower-risk alternative to traditional drug discovery approaches [9]. Epidemiological studies demonstrated improved survival in patients taking various medications for other co-morbidities and identified the anti-tumour activity of statins, bisphosphonates, metformin, aspirin, ivermectin and itraconazole in OC [10, 11]. Few repurposed drugs, such as metformin and chloroquine, have progressed in OC phase I/II clinical trials but failed to show significant clinical benefit [12, 13]. To identify more repurposed drug candidates, several high-throughput screening (HTS) with libraries of FDA-approved compounds have been conducted [14, 15]. While HTS can efficiently identify candidate compounds, many face translational challenges. For instance, milciclib was identified as a hit but was subsequently discontinued due to safety concerns. In another screen, the most potent FDA-approved compounds exhibited IC_50_ values no lower than 2 µM, which exceeds clinically achievable plasma concentrations (C_max_). These findings highlight the limitations of repurposed monotherapies, where drug combination strategies offer a compelling solution by enhancing efficacy, overcoming resistance, and allowing for dose reduction, which can improve tolerability and make otherwise non-viable compounds more clinically feasible [16].

Current approaches to high-throughput combination screening have facilitated the discovery of new cancer treatment options, identifying synergistic drug interactions involving chemotherapeutic agents or targeted inhibitors. In colorectal cancer, Folkesson et al.. explored 21 combinations of four clinical drugs with 3 targeted agents, reporting strong synergy with MEK (mitogen-activated protein kinase) inhibition [17]. In OC, several studies also explored similar pathway-guided strategies. Huang et al. conducted a drug screening combining the CHK1 (checkpoint kinase 1) inhibitor prexasertib with other compounds and identified PI3K/mTOR (phosphoinositide 3-kinase/mammalian target of rapamycin) inhibitors as potent synergistic partners that enhanced prexasertib-induced cell death [18]. Similarly, Sima et al. screened combinations with cisplatin and found that EGFR (epidermal growth factor receptor) inhibitors sensitised OC cells to it, and Noonan et al. demonstrated synergy between docetaxel and birinapant, an inhibitor of apoptosis protein (IAP) mimetic, in apoptosis-resistant OC cells [19, 20]. While these pathway-driven efforts have provided valuable insights, they are often guided by predefined biological hypotheses and focused on a limited number of molecular targets, which may overlook novel or non-canonical drug interactions, particularly in drug-resistant HGSOC, which is associated with greater complexity and disease heterogeneity [21]. To overcome these limitations, some studies adopted an unbiased approach to fully capture all possible drug interactions. For instance, Close et al. tested 45 pairings from a panel of 10 compounds in melanoma [22], while Flobak et al. screened 171 combinations of 19 inhibitors across eight cancer cell lines [23]. However, due to the exponential complexity of drug-pair matrices, most efforts remain restricted to small-scale panels of known agents, and fully unbiased combinatorial screening has not yet been applied to OC.

By integrating both approaches, we conducted an unbiased combination screening using a library of approved drugs with known safety profiles. This platform enabled efficient testing of all pairings among 384 compounds in fewer than 4,000 wells, without relying on prior mechanistic assumptions. This strategy revealed previously uncharacterised combinations with the potential to kill chemoresistant HGSOC cells that could benefit patients with treatment-resistant disease.

## Materials and methods

### Cell culture

OVCAR3, OVCAR4, and Kuramochi were kindly gifted by Dr Niamh Buckley from Queen’s University Belfast. PEO1 and PEO4 were kindly gifted by Dr Marion Butler from Maynooth University. All cell lines were authenticated by the American Type Culture Collection (ATCC). OVCAR3, OVCAR4, and Kuramochi cells were cultured in RPMI-1640 (Gibco#21875034) supplemented with 10% Fetal Bovine Serum (FBS (Sigma#F7524)). PEO1 and PEO4 were cultured in the same medium with the addition of 2 mM sodium pyruvate. All cells were cultured in a 37 °C incubator with 5% CO_2_.

### Ascites samples

The samples used in this research were received from the Northern Ireland Biobank [24], with informed consent and ethical approval (reference: NIB22-0001). Clinical details of patients, including histotype, stage, drug treatment and progression status, are summarised in Supplementary Table 1. All patients had histologically confirmed HGSOC.

### Isolation and culture of OC patients’ ascites-derived cells (PADCs)

The samples used in this research were received from the Northern Ireland Biobank [24]. Ascitic fluid was centrifuged at 3,000 rpm for 5 min at room temperature to collect the cell pellet. The pellet was washed twice with phosphate-buffered saline (PBS), and red blood cells were removed using red blood cell lysis buffer (Roche#11814389001). Primary ascites-derived cells (PADCs) were cultured in conditioned medium consisting of DMEM/F12 (Gibco #21041025) supplemented with 5% FBS, 10 ng/mL epidermal growth factor (EGF), 20 µg/mL insulin, 0.5 µg/mL hydrocortisone, and 25 ng/mL cholera toxin. The culture medium was refreshed every 3–4 days. At 95% confluency, fibroblasts were selectively removed by differential trypsinisation: cells were first treated with 0.05% trypsin to detach fibroblasts, followed by rinsing, and then 0.1% trypsin was applied to collect the remaining epithelial tumour cells. Fibroblast-like cells typically disappeared after 1–2 passages. The epithelial and HGSOC origins of the PADCs were confirmed by western blotting using specific markers (WT-1, PAX8, E-cadherin, p53). All experiments were conducted using PADCs within passages 2 to 5.

### Cytotoxicity, cell viability assays

Cytotoxicity was assessed using the CellTox Green Cytotoxicity Kit (Promega #G8741). CellTox Green dye was added to 384-well black optical bottom plates (Greiner CELLSTAR#M1937) at a 1:2000 dilution. After a 2-hour incubation, fluorescence intensity (FI) was measured using a FLUOstar Omega plate reader (BMG LABTECH, Germany) with excitation/emission at 485/520 nm. Cell viability was evaluated using the PrestoBlue Cell Viability Reagent (ThermoFisher#P50201), which was added at a 1:10 dilution and incubated for 2 h before measurement. Fluorescence was detected with excitation/emission at 544/590 nm. These assays were used in drug screening and the following assessment of copanlisib, cerivastatin, and their combinations.

To evaluate cell responses to OC standard of care treatment, including cisplatin, carboplatin, olaparib, and paclitaxel, cell viability was assessed after 72 h of treatment using the MTT assay. MTT reagent was prepared from thiazolyl blue tetrazolium bromide (ThermoFisher#L11939.06), and 0.5% MTT solution was added at a 1:10 dilution. Following a 2-hour incubation, the medium was removed, and DMSO was added to dissolve the formazan product. Absorbance was read at 570 nm using the plate reader. All drug-treatment experiments included matched vehicle controls containing 0.1% DMSO.

### Single drug screening

The single-compound screening assay was performed in 384-well black optical bottom plates (Greiner CELLSTAR#M1937) using 5 HGSOC cell lines. The initial drug library consisted of 593 compounds sourced from MedChemExpress, comprising a diverse collection of approved and investigational drugs originally developed for cancer, neurological, cardiovascular, and inflammatory diseases (Supplementary Table 2). Compounds were directly transferred to the plates without cells using an Echo 525 Acoustic liquid handler (Beckman Coulter) with desired doses (0.5, 1 and 5 µM at final cell seeding), negative control (0.1% DMSO). Cells were seeded into the wells at an optimised density to ensure 90% confluency by the end of the assay. Following 72 h of incubation in a humidified incubator at 37 °C supplemented with 5% CO_2_, several selected wells were treated with 4% lysis solution (Promega #G1821) for 15 min at room temperature to generate positive control values. Cytotoxicity of each well was then evaluated using the CellTox Green assay, and the values were normalised to the negative control.

### Z-score and Z-factor calculation

To assess the variability of drug responses within the same concentration, the Z-score was calculated using the formula below. The x represents the normalised FI values, and µ represents the mean, σ represents the standard deviation.

$$\:Z=\frac{\text{x}-{\upmu\:}}{{\upsigma\:}}$$

To assess the robustness of the HTS assay, the Z-factor was calculated for each plate [25]. A Z-factor value above 0.5 typically indicates an excellent assay [25], while values below 0.5 suggest a need for optimisation. The formula for calculating the Z-factor is as follows. $\:{\upsigma\:}\text{P}\text{O}\text{S}$ and $\:{\upsigma\:}\text{N}\text{E}\text{G}$ are the standard deviation of the positive and negative control values; $\:{\upmu\:}\text{P}\text{O}\text{S}$ and $\:{\upmu\:}\text{N}\text{E}\text{G}$ are the mean of the positive and negative control values.

$$\:{Z}^{{\prime\:}}=1-\left(\frac{3({\upsigma\:}\text{P}\text{O}\text{S}+{\upsigma\:}\text{N}\text{E}\text{G})}{|{\upmu\:}\text{P}\text{O}\text{S}-{\upmu\:}\text{N}\text{E}\text{G}|}\right)$$

### Drug sensitivity score (DSS)

The drug sensitivity score was calculated by Breeze, an online tool, with the DSS2 method and 4-parametric logic (4-PL) curve fitting, which integrates multi-dose relationships [26]. All wells were normalised to the negative control. To calculate the percentage of inhibition, the normalised value of each drug at a specific concentration was compared to the normalised values of the positive and negative controls using the following formula: %Inhibition = 100*(Nn-Nd)/(Np-Nn) where Np and Nn are the mean of normalised positive and negative values, respectively, and Nd is the normalised data.

### Multiplex screening for interacting compounds (MuSIC)

The 384 drugs that constructed the MuSIC assay were selected based on the following criteria: (1) Selection of low cytotoxic drugs with Z-Score greater than − 0.5 and less than 2 in 0.5 µM single drug screen of OVCAR3 measured by CellTox Green; (2) initiation in clinical trials; (3) elimination of analogues (Supplementary Table 3). A specialised all-pairs testing algorithm was employed to group drugs into pools of 10 compounds per well [27]. Then MuSIC was screened on OVCAR3 and Kuramochi cells with differential chemosensitivities and responses determined by PrestoBlue cell viability assay.

### Deconvolution of music into combinations screen

A 10-compounds combination was considered a hit when cell viability was inhibited over 75% measured by the PrestoBlue assay. To avoid non-specific cytotoxic effects, well-known cytotoxic drugs (e.g., bortezomib, ixazomib, melflufen, actinomycin A, idarubicin, doxorubicin) were excluded. As the MuSIC screening design was based on the single-agent response profile of OVCAR3, additional exclusions were made to account for cell line–specific sensitivity in Kuramochi cells. Specifically, any well containing a drug that exhibited more than 50% cytotoxicity in Kuramochi cells at 0.5 µM, based on the CellTox Green assay, was removed. To identify clinically feasible combinations from “hit” wells, the 10-compound combinations were deconvoluted into 45 pairwise combinations.

### Combination synergy analysis

The synergies of drug pairings were first ranked by the Bliss Independence Model. The bliss score was calculated following the formula Ebliss = Y_ab, O_ - Y_ab, P_. Y_ab, O_ is regarded as the combination effect and the predicted combined effect could be achieved by Y_ab, P_ = Y_a_ + Y_b_ - Y_a_Y_b_, where Y_a_ and Y_b_ are the observed inhibition effects of using drugs a and b alone. Effects were computed from normalised data, with upper values limited to 1. The Ebliss were categorised into strong synergism, synergism, addition, antagonism, and strong antagonism within each cell line. Each category was assigned a numerical score ranging from 2 (strong synergism) to −2 (strong antagonism) to facilitate further analysis (Fig. 3C).

Strong synergistic combinations were further validated and analysed by the Loewe additivity model. Cells were treated with a 5 × 5-dose combination matrix for 72 h at gradient concentrations, and viability was determined using PrestoBlue assay. Combenefit was used to calculate the Loewe additivity score [28]. The highest score in the matrix was used to determine synergy, where a cutoff of 10 was used to determine a potent synergistic effect, as recommended.

### Spheroid assay

Kuramochi and PEO4 cells were seeded into 96-well ultra-low attachment U-bottom plates (SARSTEDT#83.3925.400) at optimised densities of 3,000 and 5,000 cells per well, respectively, as determined by preliminary experiments. After 24 h, an equal volume of 5% Matrigel (Corning#354234) was transferred to the wells (day 1). After 96 h, spheroids were formed and treated with a serial-determined concentration of copanlisib and/or in combination with cerivastatin (day 4). 0.1% DMSO was used as a vehicle control. After 72 h of treatment, cell viability was assessed using the PrestoBlue assay, with a 2-hour incubation. For live-cell imaging, spheroids were treated on day 4 with 0.5 µM copanlisib, cerivastatin, or their combination for 72 h, followed by staining with CellTox Green Dye at a 1:2000 dilution. Images were acquired in both brightfield and green fluorescence channels, and fluorescence intensity was quantified using the Cell3iMager NX (Screen, Japan). For long-term treatment, spheroids were maintained in culture for 23 days, with treatments of 0.1 µM copanlisib, cerivastatin, or their combination administered on days 7 and 14. Spheroid growth was monitored using the Cell3iMager Neo plate imager (Screen, Japan), and size was quantified using the instrument’s pseudo-volume algorithm.

### Western blot analysis

Cell pellets were collected, washed with PBS and lysed on ice in RIPA buffer (ThermoFisher#89900) with phosphatase/protease inhibitors (CST#5872). Lysates were centrifuged at 14,000 RPM (4 °C) for 30 min, and supernatant protein concentration was quantified by modified Bradford assay (Sigma#B6916). Proteins were resolved by SDS-PAGE, transferred to membranes, and probed with primary antibodies overnight at 4 °C, followed by incubation with species-specific HRP-conjugated secondary antibodies for 1 h at room temperature. All antibodies used in this study, including dilutions and catalogue numbers, are listed in Supplementary Table 4. Blots were incubated with chemiluminescent substrate (ThermoFisher #34578) and imaged using the UVITEC gel documentation system. Raw band intensities were measured using ImageJ (NIH), and protein levels were quantified relative to loading control (β-actin or GAPDH).

### Statistical analysis

All assays were performed in at least three independent experiments, except for those involving PADCs, and data were presented with mean and standard deviation. The list of chemoresistance genes in OC was obtained from a systematic review [29], and their expression levels were extracted from DepMap [30]. Using RStudio, hierarchical clustering of drug sensitivity scores (DSS) or gene expression data across cell lines was conducted using Euclidean distance and Ward’s linkage method. The adjusted Rand index (ARI) was calculated to assess the similarity between clustering results, with values ranging from − 1 (complete disagreement) to 1 (identical clustering) [31]. Using GraphPad Prism 10.2, dose-response analysis of the treatment was calculated using the log (inhibitor) versus response (three parameters) model with a 95% confidence interval, and their significant differences were determined by the extra sum-of-squares F test. Two-way ANOVA analysis with independent factors of time and grouping was used to compare spheroid growth in long-term treatment. One-way ANOVA analysis was used to compare different treatment groups, followed by Tukey’s test. *P value < 0.05 was defined as statistically significant.

## Results

### HTS screening of HGSOC cell lines

**Fig. 1:**
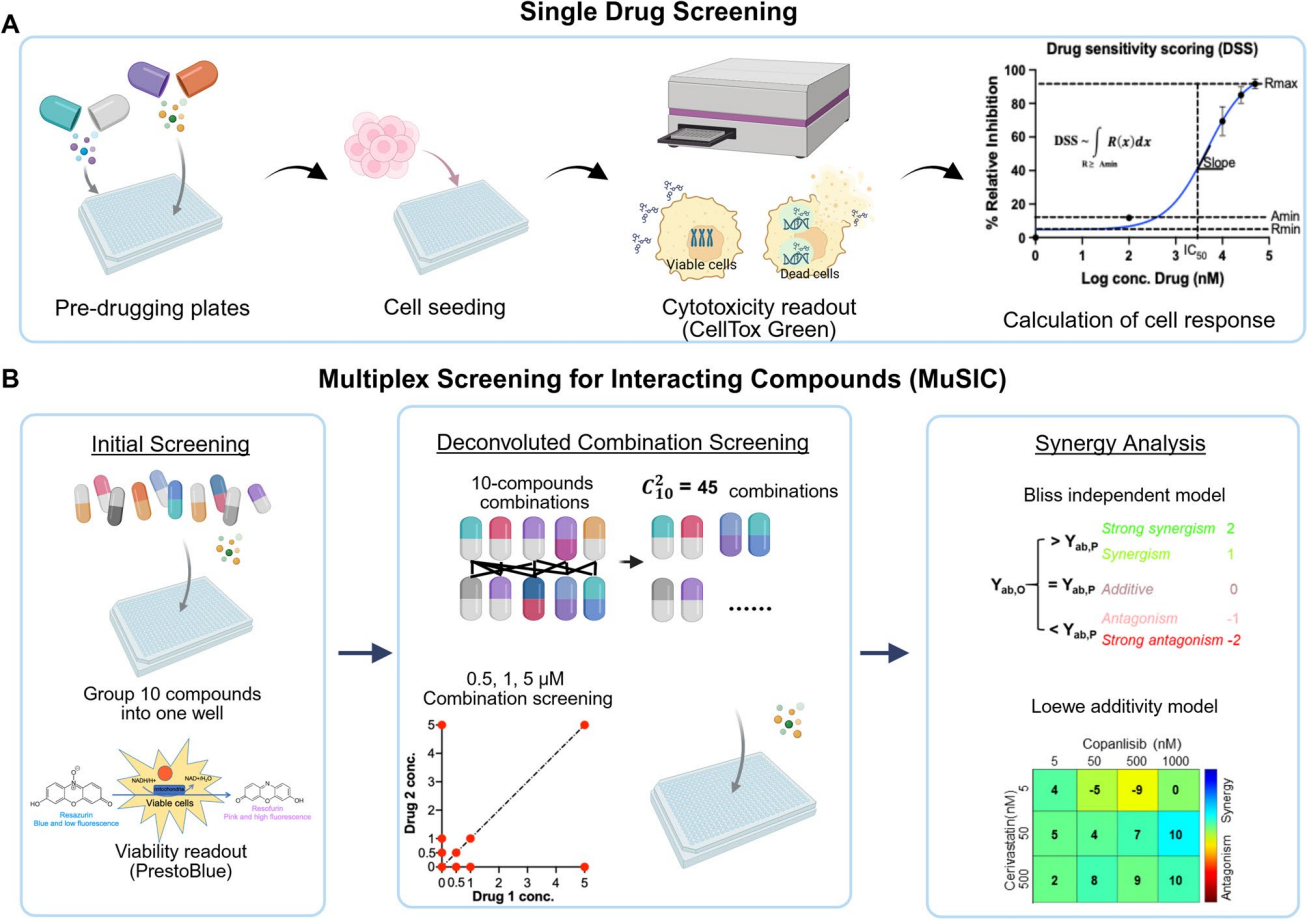
Schematic overview of the screening pipeline in HGSOC cell lines. **A** A library of 593 compounds was dispensed into 384-well plates using the ECHO 525 acoustic liquid handler, followed by cell seeding. Cytotoxicity was assessed using CellTox Green, and drug responses were quantified as DSS. **B** A subset of 384 low-cytotoxic compounds identified from the single-agent screen was pooled into 10-drug per well. Cell viability was measured using PrestoBlue. Potent pools were deconvoluted into drug pairs and synergistic interactions were first ranked by the Bliss independent model. Strong synergistic combinations were further validated by the Loewe additivity model. Figure created in https://BioRender.com

**Fig. 2:**
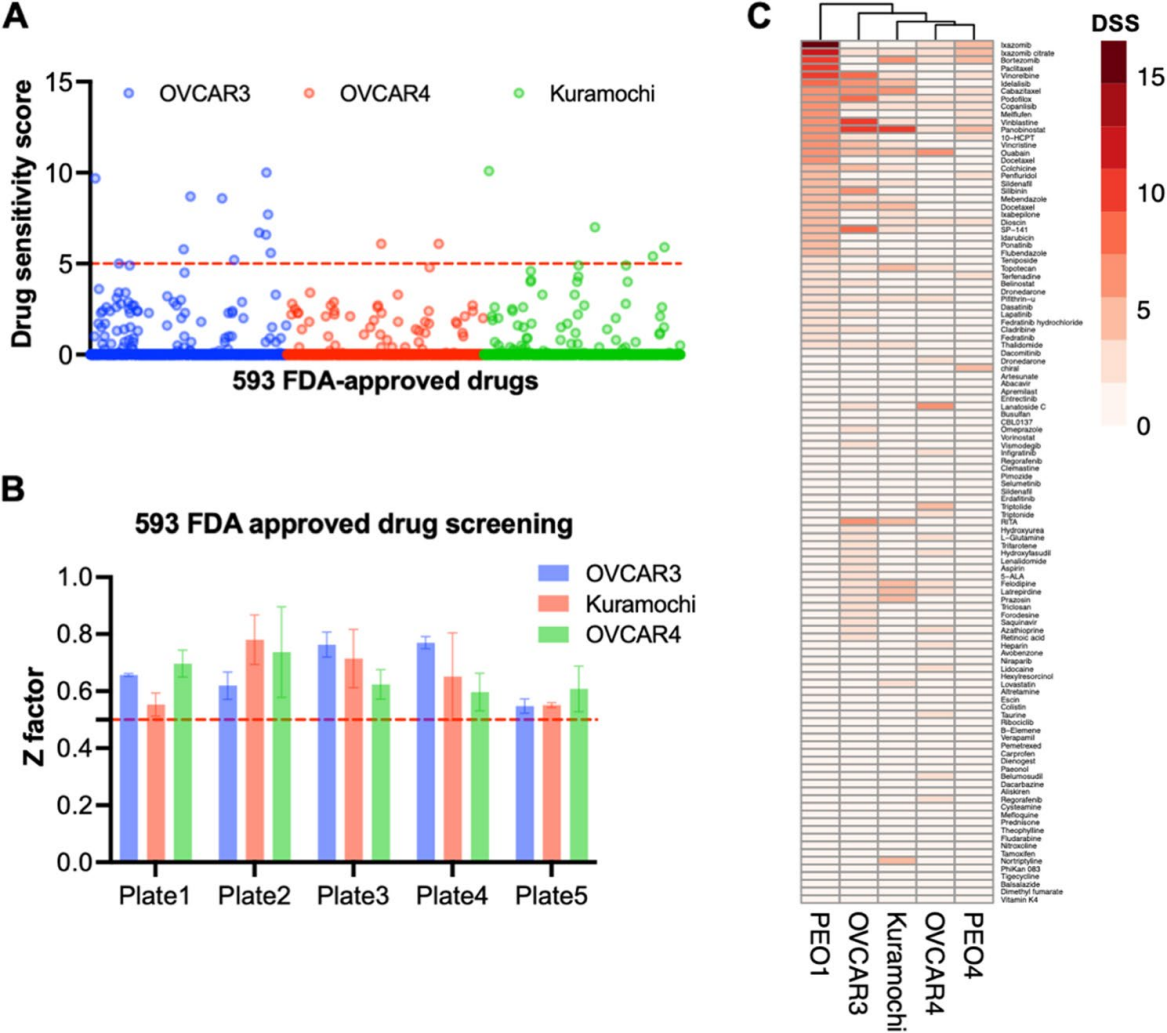
Single drug screening in HGSOC cell lines. **A** Scatter plot of all drugs’ DSS across 3 OC cell lines. The red line indicates DSS = 5, which is the cut-off for cytotoxic drugs. **B** Z-factor analysis across all plates (all > 0.5). **C** Hierarchical clustering of 5 HGSOC cell lines based on the DSS of 112 drugs

Three HGSOC cell lines (OVCAR3, OVCAR4, and Kuramochi) with distinct chemosensitivities (Supplementary Fig. 1 A and Supplementary Table 5) were screened against the initial drug library (as outlined in Fig. 1A). Cells were treated with drugs at concentrations of 0.5, 1, and 5 µM, and cell toxicity was measured at 72 h, with CellTox Green. Individual drug responses were quantified by DSS where scores > 5 implies drug sensitivity (Fig. 2A). The Z-factor analysis for each plate exceeded 0.5 (Fig. 2B). The chemosensitive OVCAR3 cell line exhibited greater drug responsiveness, with 11 compounds showing DSS > 5, compared to 4 in Kuramochi and 2 in OVCAR4 (Fig. 2A). A total of 112 drugs that demonstrated partial response (DSS > 0) across the three cell lines were further validated in the PEO1 and PEO4 cisplatin-sensitive/insensitive isogenic cell line model (Supplementary Table 6). Hierarchical clustering grouped the cell lines according to their DSS scores, which reflected their sensitivity profiles to standard-of-care chemotherapy (Fig. 2C). This suggests a correlation between chemotherapy resistance and broader drug insensitivity. We tested whether the expression of chemoresistance genes was associated with this response, however, hierarchical clustering based on the expression of chemoresistance genes (Supplementary Fig. 1B) did not align with the drug response profiles, with an ARI value of −0.06, indicating minimal correlation. Therefore, direct drug response profiling provided a stronger prediction of drug response compared to profiling the expression of chemoresistance genes. In general, the chemoresistant HGSOC cell lines responded poorly to monotherapies.

### Combinations screening in cell lines with different chemosensitivities

**Fig. 3:**
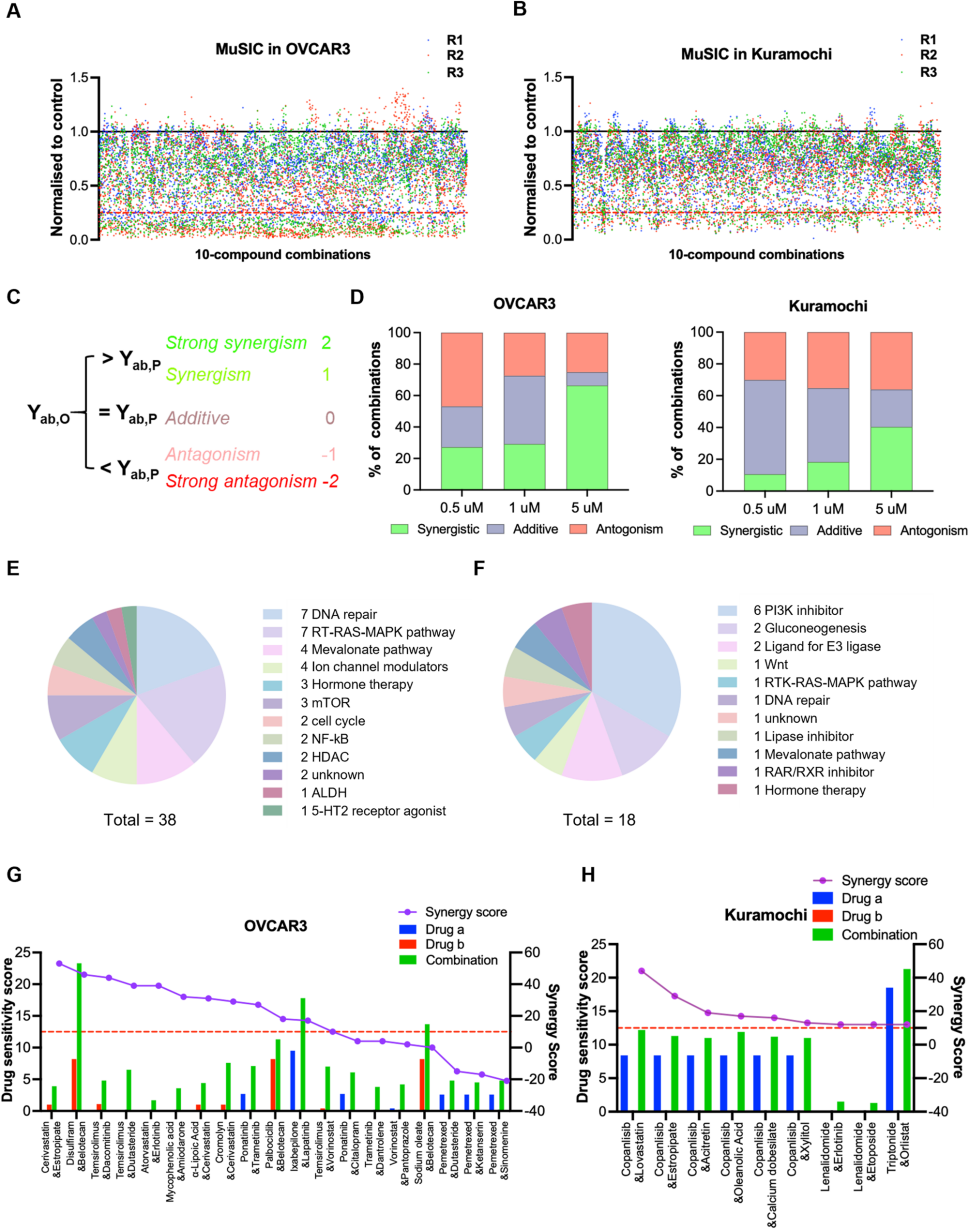
Figure 3 Multiplex screening for interacting compounds in 2 OC cell lines. **A-B** Scatter plots of normalised data points for 3878 drug combinations in OVCAR3 and Kuramochi. **C** Bliss model analysis of drug combination effects, with Yab,O and Yab,P representing observed and predicted inhibition rates, respectively. Synergy scores range from 2 (synergistic) to -2 (antagonistic). **D **Stacked bar graph showing the distribution of synergistic, additive, and antagonistic combinations in OC cell lines, as assessed by the Bliss independence model. **E-F** Pie charts of targets that involved in strong synergistic combinations in OVCAR3 (**E**) and Kuramochi (**F**). **G-H** Comparison of DSS values and synergy scores for drug combinations in OVCAR3 (**G**) and Kuramochi (**H**). The bar of blue, red and green represented drug a and b and their combination, respectively and the purple dot line represented the synergy score calculated by the Loewe additivity model

To identify cytotoxic drug combinations which specifically treat chemoresistant cells, we employed MuSIC to evaluate all conceivable pairwise combinations of 384 drugs (as outlined in Fig. 1B). The screening was performed in two HGSOC cell lines at a fixed concentration of 0.5 µM per compound. Although cells were treated with 10-compound combinations (total concentration of 5 µM), Kuramochi exhibited fewer responsive combinations (3.76%) compared to OVCAR3 (11.76%) (Fig. 3A and B). Each cytotoxic 10-compound combination was deconvoluted to test all drug pairings. The synergy score for each drug pairing was calculated using the Bliss independent model, assigning scores from − 2 (strong antagonistic) to 2 (strong synergistic) (Fig. 3C). More synergistic combinations appeared as the dose increased. The OVCAR3 cell line was sensitive to more synergistic combinations compared to Kuramochi, which corroborated the results produced by the MuSIC screen (Fig. 3D). Moreover, drug targets that were involved in strong synergistic combinations revealed a distinct pattern between the two cell lines, where OVCAR3 cells were more sensitive to drug combinations involving traditional anticancer targets such as DNA targeting agents and inhibitors of RAS/MAPK pathway while Kuramochi cells were only sensitive to drug combinations involving PI3K inhibition (Fig. 3E and F).

Strong synergistic combinations were further assessed by two more mathematical models for drug combinations. By simply comparing the DSS scores between single agents and their combination, the DSS for all potent combinations were higher than their single agents. However, during Loewe additive model analysis, only 11 out of 19 pairings produced synergy scores above 10 in the OVCAR3 cells (Fig. 3G). All 9 combinations tested in the Kuramochi cells passed the assessing criteria, although the difference between the combination and single agent DSS was minor (Fig. 3H). The variation between synergy calculation models indicated that the synergistic effect only appeared at a specific dose pair level [32]. However, our goal was to identify synergistic drug pairs with translational potential. We therefore focused on the 0.5 µM combination screen, selecting a concentration generally below or within the range of reported C_max_ for many approved drugs. The cytotoxicity responses for each drug combination were further investigated on the isogenic PEO1 and PEO4 cells.

### Copanlisib synergises with cerivastatin across chemoresistant cell lines

**Fig. 4:**
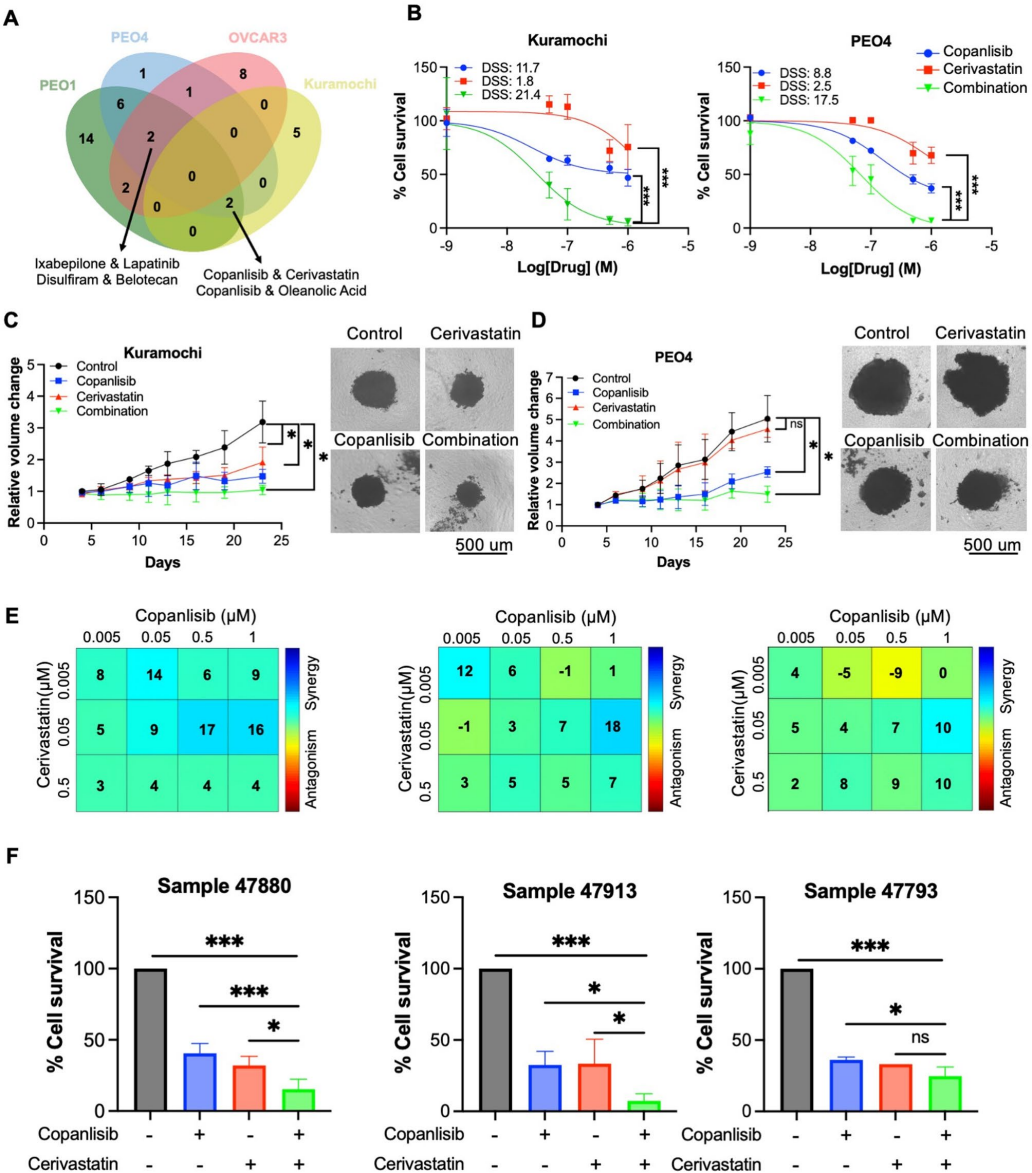
Synergistic effect of copanlisib and cerivastatin validated in chemoresistant OC cells and PADCs. **A** Venn diagram showing potent drug combinations across four OC cell lines at 0.5 µM. **B** Dose-response curve analyses for Kuramochi and PEO4 treated with copanlisib, cerivastatin, and copanlisib in combination with 0.5 µM cerivastatin in 2D culture for 72 h. Cell viability was measured by PrestoBlue assay. **C-D** Spheroid growth of Kuramochi (**C**) and PEO4 (**D**) treated with copanlisib, cerivastatin, and copanlisib in combination with 0.1 µM for 18 days, with representative graphs at day 23. **E** Loewe additivity model of synergy profiles for copanlisib and cerivastatin in three PADCs. **F** Bar chart of copanlisib and cerivastatin in three PADCs treated with 0.5 µM copanlisib and 0.05 µM cerivastatin and in combination. Experiments of PADCs were conducted at *n* = 2 with technical duplicates. Scale bar: 500 nm. * *P* < 0.05, **P < 0.01, ***P < 0.001

From the 0.5 µM combination screen across OVCAR3, Kuramochi, PEO1, and PEO4, four combinations were consistently identified in at least three cell lines, showing >50% inhibition and classified as strongly synergistic: copanlisib with cerivastatin or oleanolic acid, belotecan with disulfiram, and ixabepilone with lapatinib (Fig. 4A and Supplementary Table 7). Notably, the combination of ixabepilone and lapatinib has already entered a phase I clinical trial for advanced breast cancer [33], highlighting the robustness of the drug screening approach employed in this study. The copanlisib and cerivastatin combination was prioritised for further investigation based on its consistently high synergy scores across resistant cell lines (Supplementary Fig. 2A).

Increased drug sensitivity to this combination was observed in both 2D and 3D cultures of Kuramochi and PEO4 cells. Dose–response curve analysis revealed leftward and downward shifts in the combination curves compared to either monotherapy (Fig. 4B and Supplementary Fig. 2B), indicating a synergistic effect. This combination also significantly amplified cytotoxicity in both 2D and 3D spheroid cultures, as measured by green fluorescence (Supplementary Fig. 2C). While spheroids treated with either copanlisib or cerivastatin alone displayed partial fluorescence, those treated with the combination exhibited near-complete fluorescence throughout the structure, indicating widespread cell death (Supplementary Fig. 2D and E). Spheroid growth was also significantly suppressed under combination treatment over a 23-day period following twice-weekly dosing (Fig. 4C and D).

To further investigate the potential of copanlisib and cerivastatin, we tested primary ascites-derived cancer cells (PADCs) to better mimic in vivo conditions. All PADCs were derived from advanced HGSOC patients who had undergone systematic anti-cancer therapy (Supplementary Table 1). Their origins were confirmed by morphological assessment and western blot analysis for E-cadherin, WT1, PAX8 and p53 (Supplementary Fig. 3A). Ex vivo drug treatment revealed poor responses to monotherapy with carboplatin, as well as limited effects from copanlisib and paclitaxel. Notably, all patient samples showed high sensitivity to cerivastatin alone (Supplementary Fig. 3D and E). Based on this, the dose of cerivastatin was reduced in subsequent experiments to validate its synergy with copanlisib in PADCs. Synergy was calculated using the Loewe additivity model. High synergy scores (Loewe > 10) were observed in samples 47880 and 47913, while sample 47 793 exhibited an additive effect (Loewe = 10) (Fig. 4E). The combination of 0.5 µM copanlisib and 0.05 µM cerivastatin produced an additional 20% reduction in cell viability compared to monotherapies in two out of three samples (Fig. 4F). Together, these findings suggested that copanlisib and cerivastatin exert synergistic effects in chemoresistant HGSOC models, including primary cultures derived from the ascites of chemo- and drug-refractory end-stage OC patients.

### Cerivastatin sensitised chemoresistant cells to copanlisib by upregulating phospho-Akt

**Fig. 5:**
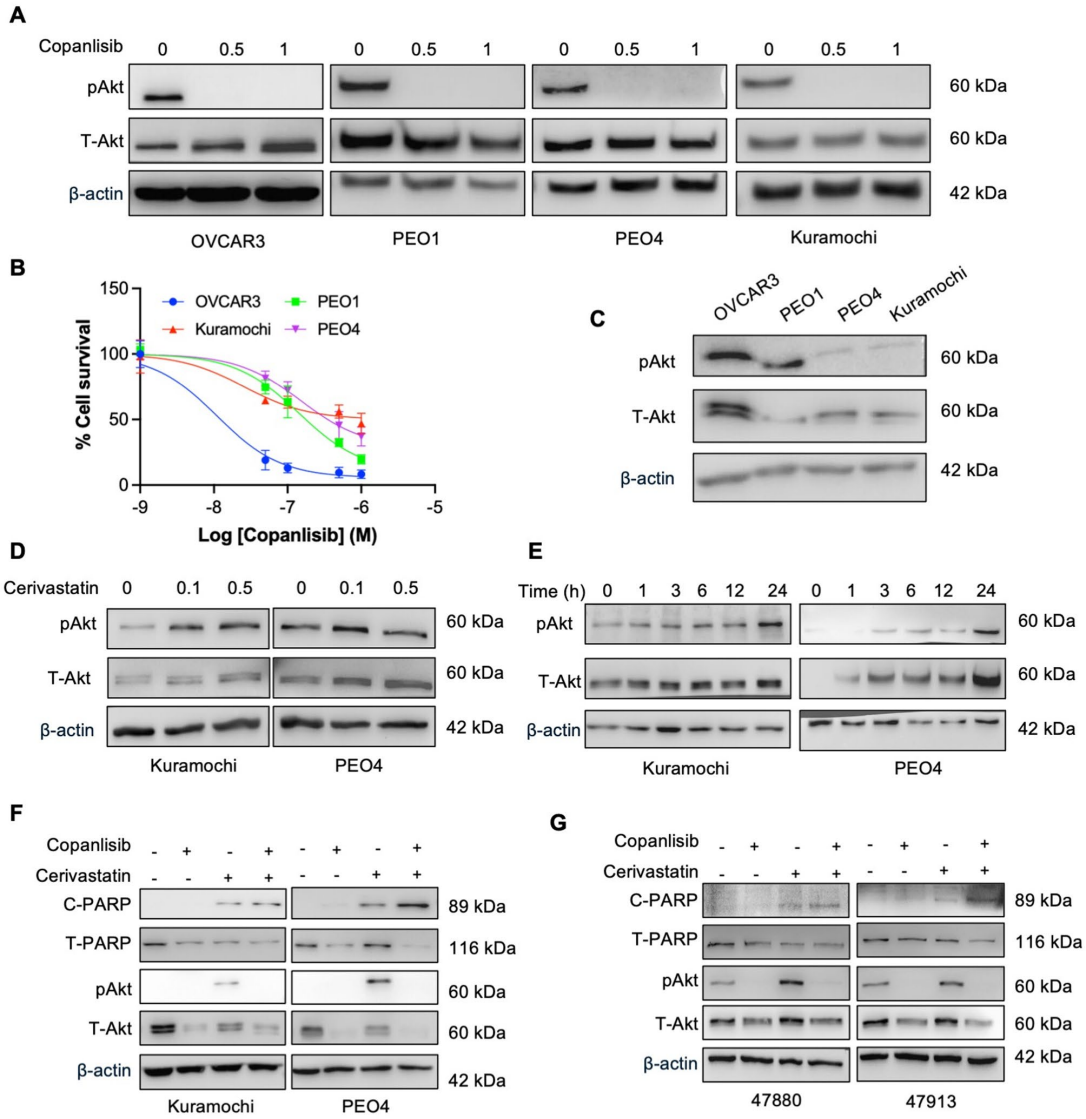
Synergistic interaction of copanlisib and cerivastatin in chemoresistant OC cells via increased phospho-Akt levels. **A** WB analysis of pAkt/T-Akt level after copanlisib 0.5 and 1 µM, 0.1% DMSO treatment for 24 h in OVCAR3, PEO1, PEO4 and Kuramochi cells. **B** Dose-response curves for copanlisib in OVCAR3, Kuramochi, PEO1 and PEO4 cell lines. **C** WB analysis of pAkt/T-Akt level across OVCAR3, PEO1, PEO4, and Kuramochi cell lines. **D** WB analysis of pAkt/T-Akt level after cerivastatin 0.1 and 0.5 µM, 0.1% DMSO treatment for 24 h in Kuramochi and PEO4 cells. **E** WB analysis of pAkt/T-Akt level during time course treatment (0–24 h) of cerivastatin at 0.5 µM in Kuramochi and PEO4 cells. **F** WB analysis of cleaved/total PARP (C-PARP/T-PARP) and pAkt/T-Akt level after copanlisib (0.5 µM), cerivastatin (0.5 µM) and their combination (0.5 µM) for 48 h in Kuramochi and PEO4 cells. **G** WB analysis of C-PARP/T-PARP and pAkt/T-Akt level after copanlisib (0.5 µM), cerivastatin (0.05 µM) and their combination for 24 h in cells derived from sample 47880 and 47913

As copanlisib is a pan-class I PI3K inhibitor and its target activity was validated by a reduced level of phospho-Akt (pAkt) in clinical trials [34], we first examined its targeting activity. Treatment with 0.5 µM copanlisib decreased pAkt levels in all four OC cell lines tested (Fig. 5A and Supplementary Fig. 4A). Dose–response analysis showed that the two chemosensitive cell lines were more sensitive to copanlisib monotherapy, with 1 µM copanlisib producing ~ 80% loss of viability in the chemosensitive OVCAR3 and PEO1 cells compared to a 50% loss of viability in the chemoresistant Kuramochi and PEO4 cells (Fig. 4B). This differential response may be explained by higher basal pAkt levels (Fig. 4C) and pathway alterations, as DepMap data indicate OVCAR3 harbours a PIK3R1 loss-of-function mutation, PEO1/PEO4 carry non-disruptive PIK3CD mutations, and Kuramochi lacks mutations in key PI3K genes. These features are consistent with clinical findings that copanlisib is more effective in tumours with PI3K-pathway alterations and elevated pAkt [34, 35].

Given the reduced efficacy of copanlisib in tumours with low basal pAkt, it was unclear why drug combinations involving copanlisib would be cytotoxic in the HGSOC cell lines. Unexpectedly, an increase in phosphorylated and total Akt (pAkt/T-Akt) levels was observed in chemoresistant HGSOC cell lines following 24 h of treatment with cerivastatin (Fig. 5D and Supplementary Fig. 4B). Upregulation of pAkt appeared as early as 1-hour post-treatment with cerivastatin in Kuramochi and PEO4 cells, with the highest detection measured at 24 h of cerivastatin treatment (Fig. 5E and Supplementary Fig. 4C). Notably, co-treatment with copanlisib and cerivastatin for 48 h resulted in complete suppression of pAkt, accompanied by elevated levels of cleaved PARP (C-PARP) in both cell lines, suggesting enhanced cytotoxicity occurred through inhibition of Akt signalling (Fig. 5F and Supplementary Fig. 4D). Similar effects were observed in two patient-derived ascites cultures showing a synergistic effect, where combined treatment with 0.5 µM copanlisib and 0.05 µM cerivastatin for 24 h also led to loss of pAkt with the emergence of PARP cleavage (Fig. 5G).

To assess whether this synergy was specific to cerivastatin, we conducted preliminary experiments using lovastatin, a clinically approved statin with a better safety profile. Lovastatin co-treatment similarly enhanced the cell response to copanlisib by shifting the dose–response curve leftward and downward, and increasing DSS values compared to monotherapy (Supplementary Fig. 5A). Like cerivastatin, lovastatin treatment also resulted in elevated pAkt levels (Supplementary Fig. 5B and C), indicating a potential shared mechanism of pathway modulation. These findings suggest that statin-induced Akt activation may sensitise chemoresistant HGSOC cells to copanlisib, potentially expanding its use to tumours lacking PI3K-pathway mutations or with low basal pAkt levels.

## Discussion

Drug repurposing using pre-printed compound libraries offers a powerful platform for rapidly identifying candidate therapeutics for diseases with unmet clinical needs, including HGSOC. The success rate of drug repurposing programmes could be higher by incorporating key recommendations from previous studies [36], such as using disease-related models [37] and de-risked drug libraries with established preclinical safety data [38]. However, current drug screens in HGSOC have relied on poorly representative models such as A2780 and SKOV3 or concentrated on single-compound screens [19, 39, 40], potentially limiting translational relevance. To address these issues, we conducted HTS with a repurposed drug library on cell models which represent the gene mutation and expression profiles associated with HGSOC [40].

The drug repurposing screen of single agents identified several effective compounds (DSS ≥ 5 in two cell lines) that had previously been studied in OC, including bortezomib (a proteasome inhibitor) [41], panobinostat (a pan-HDAC inhibitor) [42], and several mitosis inhibitors [43] (Supplementary Table 2), supporting the robustness of the single drug screen presented in this study. A small panel of sensitive drugs was used to cluster cell lines, which grouped cells according to their chemoresponse profile. Notably, the isogenic pair PEO1 and PEO4, derived from the same patient before and after developing platinum resistance, exhibited markedly different responses not only to platinum agents but also to a broader panel of compounds (Fig. 2C), suggesting that acquired platinum resistance may be associated with multidrug resistance [44]. However, the expression of canonical chemoresistance-related genes was more similar between PEO1 and PEO4 than with other lines, and did not cluster according to drug sensitivity, indicating that additional, non-transcriptional mechanisms may contribute to the resistant phenotype.

In HGSOC, resistance gradually emerges across a broad range of therapeutic agents, including PARP inhibitors, anti-angiogenic therapies, and immune checkpoint inhibitors, aligning with our single-drug screening results and previous in vitro studies [45–48]. Although several clinical trials have evaluated rational combinations, such as checkpoint inhibitors with alkylating agents or PARP inhibitors [5, 49], clinical benefit has generally been modest and restricted to small patient subsets [50], likely reflecting the high genomic instability and low prevalence of actionable mutations in HGSOC [51]. Given the limited success of monotherapies and the scarcity of effective combination options, we employed an unbiased MuSIC-based screening method [27] that does not rely on predefined pathways, enabling efficient evaluation of thousands of drug pairs with reduced assay burden (3,878 vs. >73,000 wells). Differential responses were observed between chemosensitive and chemoresistant cells. OVCAR3 cells responded to drug pairs targeting DNA repair and microtubules, while chemoresistant Kuramochi cells were more responsive to combinations involving PI3K inhibition. By leveraging existing drugs with known safety profiles, this strategy offers a rapid and translationally relevant route to identify new treatment options that may improve outcomes for women with HGSOC.

Among the identified pairings at 0.5 µM, we focused on copanlisib and cerivastatin, which consistently exhibited strong synergy at low concentrations in chemoresistant HGSOC models, making them a promising candidate for further investigation. Copanlisib, a pan-class I PI3K inhibitor, was approved by the FDA in 2017 for relapsed follicular lymphoma but was later withdrawn after the Phase III CHRONOS-4 trial failed to show additional benefit over immunochemotherapy [52, 53]. In solid tumours, activity has been observed mainly in those harbouring PI3K-pathway alterations [54], consistent with our data showing weak responses in cell lines without active PI3K signalling and PADCs, likely reflecting the low frequency of such alterations in HGSOC (~ 2.3–3% PIK3CA mutation, ~ 18% AKT2 amplification) [55, 56]. To date, only one study (NCI10217) has reported combining olaparib with copanlisib in one ovarian cancer patient, but no results are currently available for this trial [57], and two trials in lymphoma have evaluated copanlisib with venetoclax [58, 59].

In this study, we showed that cerivastatin sensitised chemoresistant HGSOC cells and PADCs to copanlisib. Statins were originally developed to lower cholesterol through regulating the mevalonate (MVA) pathway, and epidemiology studies in OC showed they can improve overall survival from 28.8 to 32.3 months [60, 61]. Although in vitro studies have demonstrated their anti-proliferative and pro-apoptotic effects in multiple cancer models, including ovarian cancer (OC), their cytotoxic activities were observed under high concentrations (>5 µM) [62, 63], limiting their translational potential. Our results demonstrated strong synergy with copanlisib at nanomolar doses in HGSOC cell lines. Moreover, all chemoresistant PADCs demonstrated sensitivity to this drug, with IC_50_ below 50 nM, suggesting a vulnerability of HGSOC to lipid signalling and cholesterol homeostasis. This is likely governed by the MVA pathway, which has been implicated in multidrug resistance through cholesterol-dependent P-glycoprotein activity [64], supporting the rationale for statin-based combinations.

Interestingly, cerivastatin treatment increased pAkt levels in chemoresistant cells, in contrast to previous cancer studies reporting pAkt suppression at higher concentrations (5–10 µM) [63, 65]. Our findings are more consistent with low-dose effects seen in non-malignant cells, where statins such as simvastatin have been shown to upregulate pAkt at ~ 1 µM [66–68]. A similar effect was observed with lovastatin in chemoresistant HGSOC lines and with cerivastatin in PADCs, both at concentrations below their IC_50_ values. Several potential mechanisms might underlie paradoxical pAkt activation, including impaired PTEN activity via reduced RhoA prenylation [69], cholesterol depletion-induced PI3K/Akt signalling [70], or mild ER stress activating compensatory survival pathways [71]. Notably, the cerivastatin-induced activation of pAkt was abrogated by co-treatment with copanlisib, leading to reduced cell viability, which was more pronounced in cells with low basal pAkt levels. Given that HGSOC often has a low mutation rate in the PI3K/Akt pathway and shows limited response to copanlisib alone, combining copanlisib with cerivastatin could represent a promising strategy to improve outcomes in cancers with inherently low pAkt levels.

Of note, cerivastatin was withdrawn in 2001 after being linked to rhabdomyolysis, a severe muscle-degenerating condition that led to kidney failure and death, with 31 fatalities reported in the United States (~ 700,000 users) and 21 worldwide [72]. The incidence of fatal rhabdomyolysis was 10-fold higher than with other statins, particularly during long-term use (~ 9 months) or with concomitant gemfibrozil, where the rate increased from 3/100,000 with monotherapy to 66/100,000 [73]. Despite these concerns, cerivastatin is ~ 100-fold more potent than other statins, and its synergy with copanlisib occurred at nanomolar concentrations, suggesting that therapeutic benefit may be achievable under short-term, carefully monitored oncology regimens [74]. Moreover, lovastatin was synergised with copanlisib in our study, but only at concentrations above its Cmax, underscoring that cerivastatin’s superior potency enables synergy at clinically relevant doses. Together, these findings support further study of cerivastatin–copanlisib combinations in oncology and highlight the need to identify alternative statins with both high potency and safer clinical profiles.

In conclusion, MuSIC represents a powerful, unbiased approach for identifying effective and synergistic therapeutic strategies. In this study, the combination of copanlisib and cerivastatin emerged as a promising candidate for repurposing in treating drug-resistant HGSOC. Expanding this screening approach with larger drug libraries and clinically relevant models could reveal more undefined drug interactions and novel synergistic pathways for overcoming resistance phenotypes in HGSOC.

## Supplementary Information

Supplementary Material 1: Supplementary Table 1: Clinical characteristics of HGSOC patients providing ascites for PADC cultures. Supplementary Table 2: DSS of 593 compounds across OVCAR3, OVCAR4, and Kuramochi cell lines. Supplementary Table 3: Compounds included in the MuSIC screening library with corresponding single-drug Z-scores in OVCAR3 cells. Supplementary Table 4: List of Primary and secondary antibodies for WB. Supplementary Table 5: DSS of OC standard-of-care chemotherapy drugs across 5 HGSOC cell lines. Supplementary Table 6: DSS of 112 selected compounds across five HGSOC cell lines. Supplementary Table 7: Potent drug combinations identified at 0.5 µM across 4 HGSOC cell lines.

Supplementary Material 2: Supplementary Fig. 1: Chemo drug response and chemoresistance genes profiling in 5 OC cell lines. Supplementary Fig. 2: Short-term treatment of copanlisib and cerivastatin in chemoresistant OC cells. Supplementary Fig. 3: Characterisation of patient ascites-derived cells. Supplementary Fig. 4: Semi-quantification of WB analysis of copanlisib, cerivastatin and combination treatment. Supplementary Fig. 5: Synergistic effect of copanlisib and lovastatin validated in chemoresistant OC cells.

## Data Availability

No datasets were generated or analysed during the current study.
