# Supplementary material for: Unbiased combination screening on repurposed drugs reveals synergistic potential of copanlisib and cerivastatin against chemoresistant high-grade serous ovarian cancer
Source: J Ovarian Res. 2025 Nov 6;18:242. doi: 10.1186/s13048-025-01828-7 (PMC12593923; doi:10.1186/s13048-025-01828-7)
Supplement: Supplementary file 2 — Supplementary Material 2: Supplementary Fig. 1: Chemo drug response and chemoresistance genes profiling in 5 OC cell lines. Supplementary Fig. 2: Short-term treatment of copanlisib and cerivastatin in chemoresistant OC cells. Supplementary Fig. 3: Characterisation of patient ascites-derived cells. Supplementary Fig. 4: Semi-quantification of WB analysis of copanlisib, cerivastatin and combination treatment. Supplementary Fig. 5: Synergistic effect of copanlisib and lovastatin validated in chemoresistant OC cells. [file 13048_2025_1828_MOESM2_ESM.docx]

**Supplementary Figures**

**
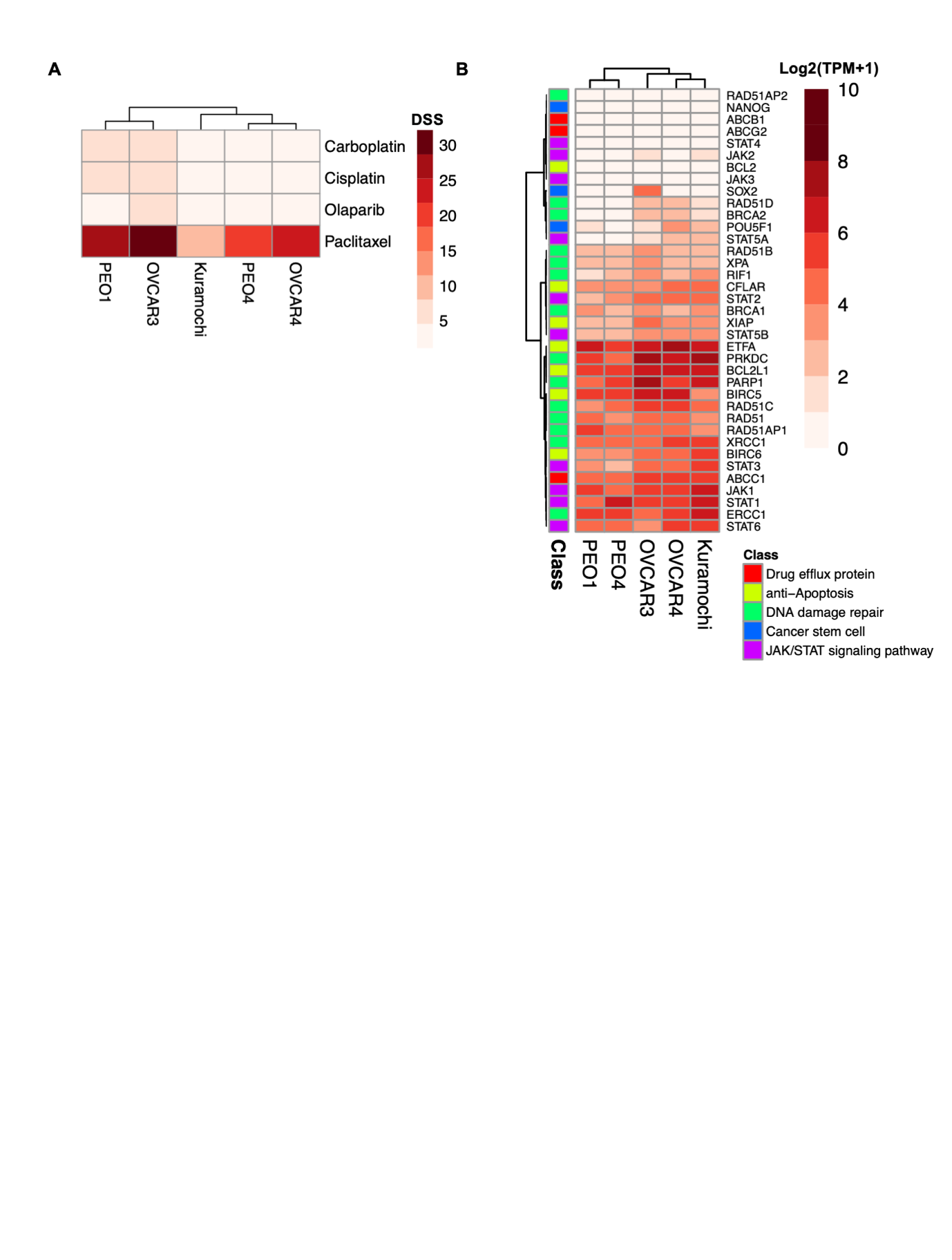
**

**Supplementary Figure 1 Chemo drug response and chemoresistance genes profiling in 5 OC cell lines.** A. Heatmap of 5 HGSOC cell lines based on the DSS of ovarian cancer standard chemotherapy drugs calculated from MTT assay. B. Heatmap of 5 HGSOC cell lines based on the chemoresistance-related genes (DepMap).

**
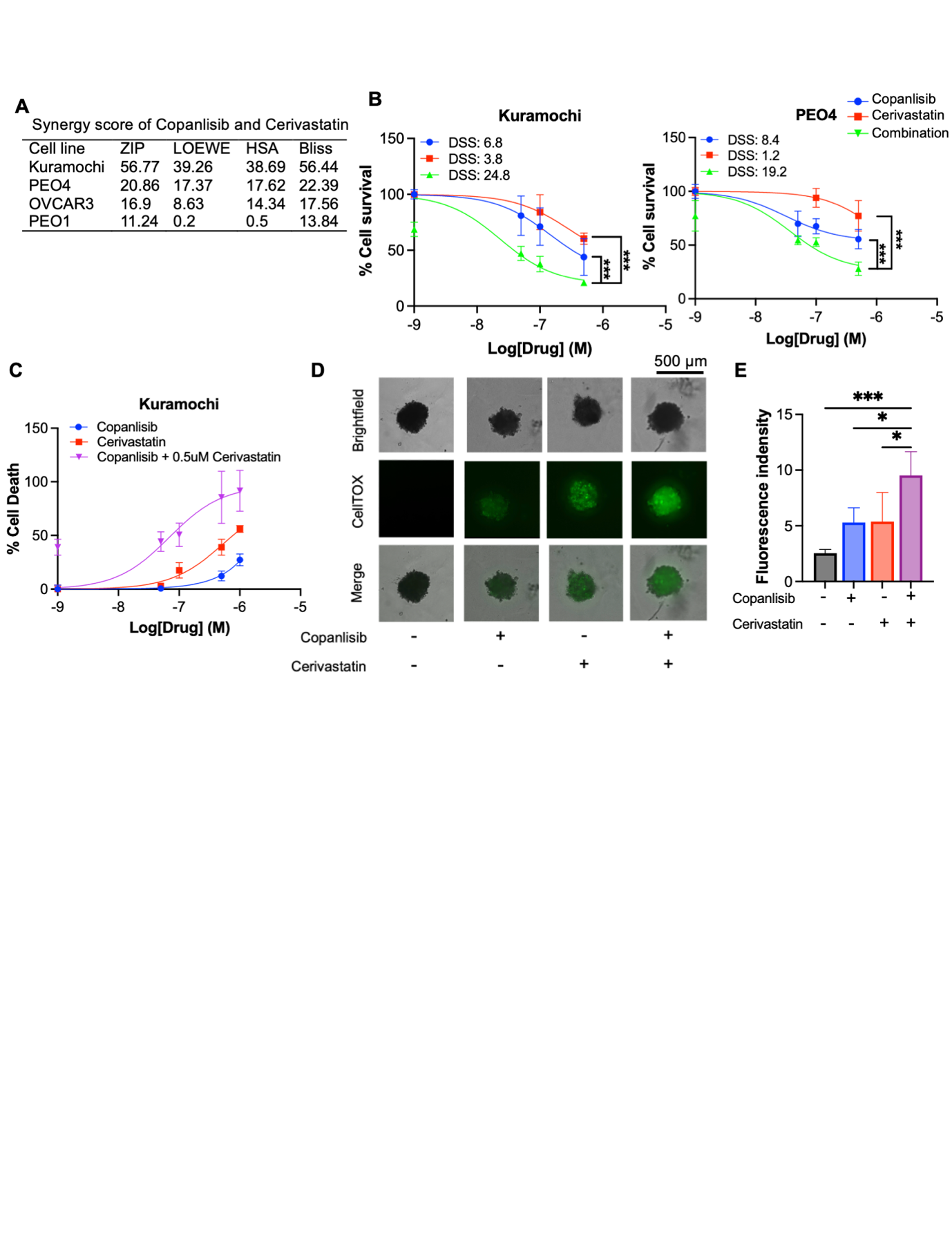
**

**Supplementary Figure 2 Short-term treatment of copanlisib and cerivastatin in chemoresistant OC cells.** A. Table summarising the highest synergy scores for copanlisib and cerivastatin combinations, calculated using various synergy models. B. Dose-response curve analyses for Kuramochi and PEO4 treated with copanlisib, cerivastatin, and copanlisib in combination with 0.5 μM cerivastatin in spheroid culture for 72 hours. Cell viability was measured by PrestoBlue assay. C. Cytotoxicities of 72 hours treatment of copanlisib, cerivastatin, and copanlisib in combination with 0.5 μM cerivastatin in Kuramochi 2D culture. D. Representative images of spheroids in Kuramochi treated with copanlisib, cerivastatin, and their combination at 0.5 μM for 72 hours, showing green fluorescence and brightfield merged views (scale bar: 500 μm). E. Quantified green fluorescence intensity of Kuramochi spheroids treated as shown in panel D. ** P < 0.05, **P < 0.01, ***P < 0.001.*

**
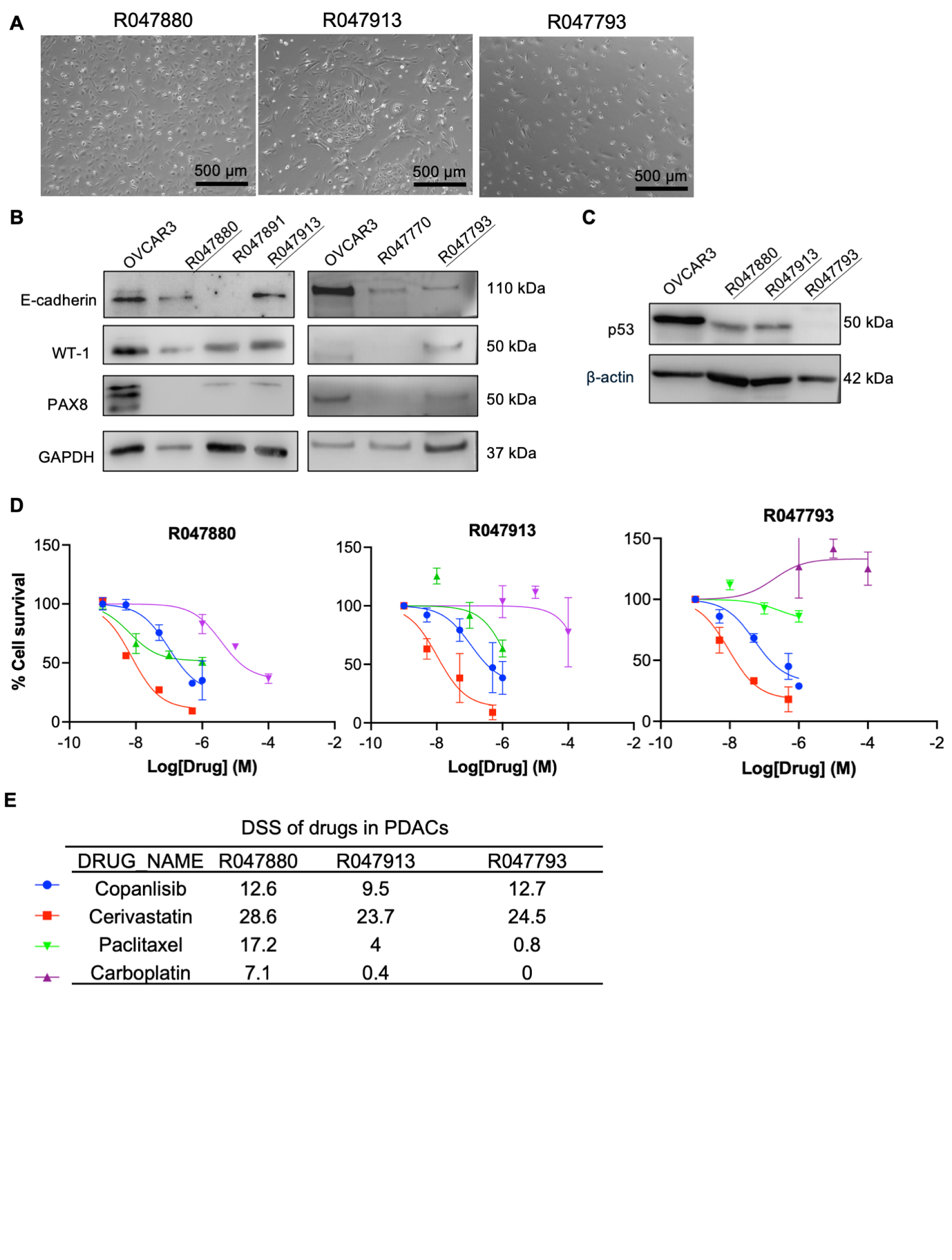
Supplementary Figure 3 Characterisation of patient ascites-derived cells.** A. Phase-contrast images of sample R047880, R047913 and R047793 at passage 1 (scale bar: 500 μm). B. WB analysis of E-cadherin, WT1, PAX8 in OVCAR3, primary cells derived from sample R047880, R047891, R047913, R047770, R047793. GAPDH was used as the loading control. C. WB analysis of p53 in OVCAR3, primary cells derived from samples R047880, R047913, and R047793. β-actin was used as the loading control. D. Dose-response curve analysis of copanlisib and cerivastatin in three PADCs (n=2 biological repeats with technical duplicates). E. Table summarising the DSS of drugs in three PADCs.


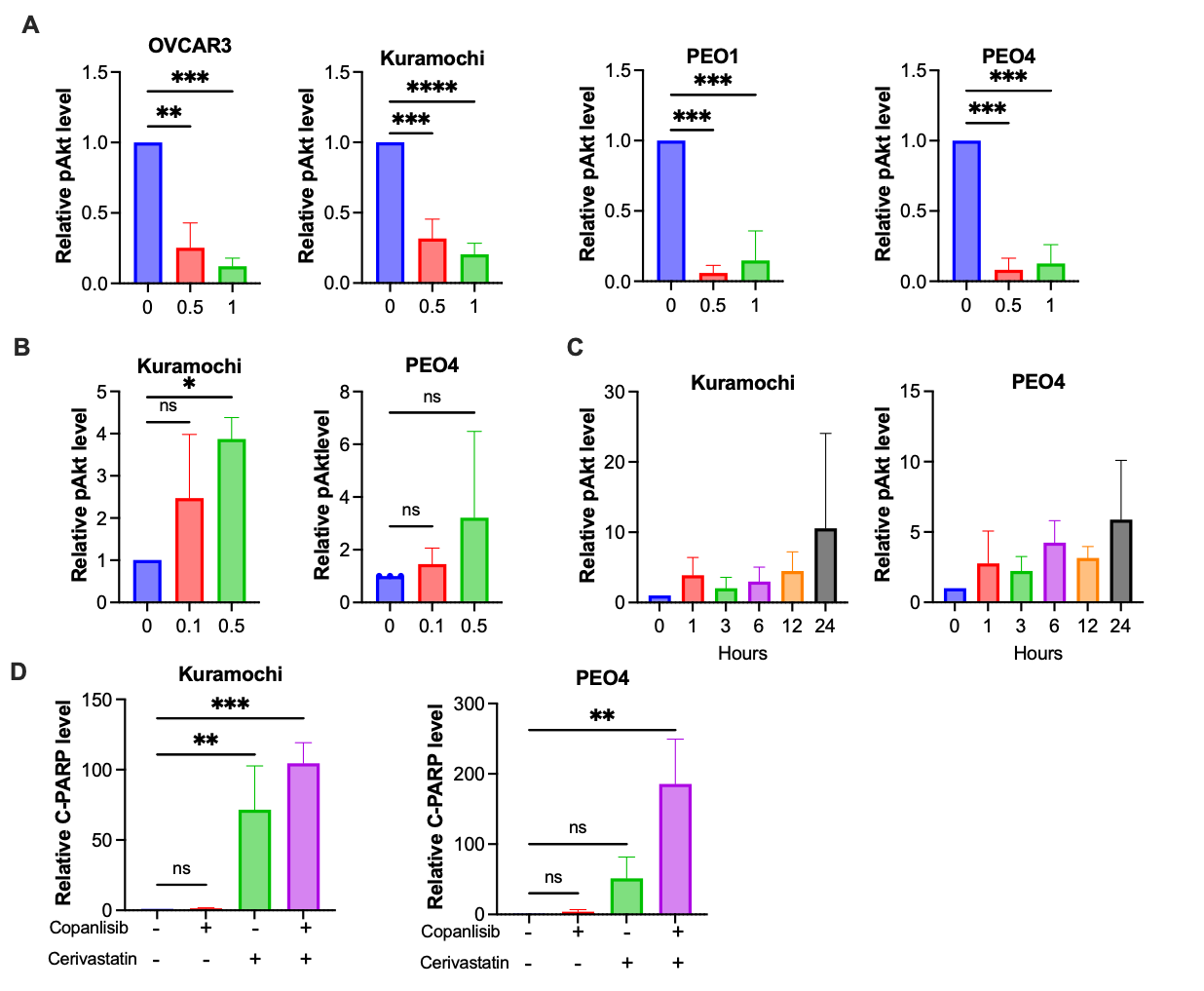


**Supplementary Figure 4 Semi-quantification of WB analysis of copanlisib, cerivastatin and combination treatment.** A. Semi-quantification of pAkt level after copanlisib 0.5 and 1 μM, 0.1% DMSO treatment for 24 hours in OVCAR3, Kuramochi, PEO1 and PEO4 cells. B. Semi-quantification of pAkt level after cerivastatin 0.1 and 0.5 μM, 0.1% DMSO treatment for 24 hours in Kuramochi and PEO4 cells. C. Semi-quantification of pAKT level during time course treatment (0-24h) of cerivastatin at 0.5 μM in Kuramochi and PEO4 cells. D. Semi-quantification of cleaved-PARP level after copanlisib (0.5 μM), cerivastatin (0.5 μM) and their combination (0.5 μM) for 48 hours in Kuramochi and PEO4 cells. * *P < 0.05,* ***P < 0.01,* ****P < 0.001*.


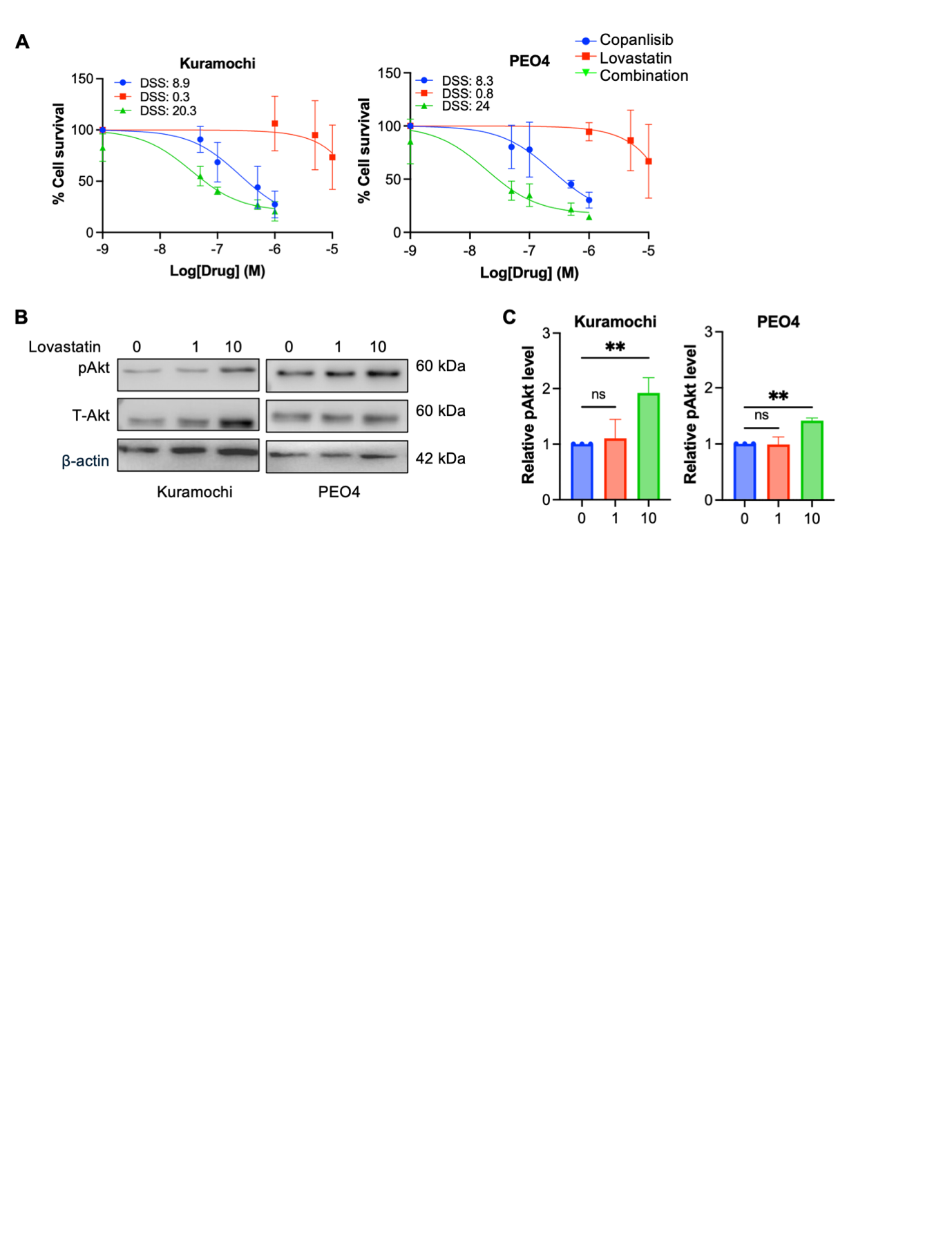


**Supplementary Figure 5 Synergistic effect of copanlisib and lovastatin validated in chemoresistant OC cells.** A. Dose-response curve analyses for Kuramochi and PEO4 treated with copanlisib, lovastatin, and copanlisib in combination with 10 μM lovastatin in 2D culture for 72 hours. Cell viability was measured by MTT assay. B. WB analysis of pAkt/T-Akt level after lovastatin 1 and 10 μM, 0.1% DMSO treatment for 24 hours in Kuramochi and PEO4 cells. C. Semi-quantification of pAkt level after lovastatin 1 and 10 μM, 0.1% DMSO treatment for 24 hours in Kuramochi and PEO4 cells. * *P < 0.05,* ***P < 0.01*.
